# Stable Isotope Analysis of Precipitation Samples Obtained via Crowdsourcing Reveals the Spatiotemporal Evolution of Superstorm Sandy

**DOI:** 10.1371/journal.pone.0091117

**Published:** 2014-03-11

**Authors:** Stephen P. Good, Derek V. Mallia, John C. Lin, Gabriel J. Bowen

**Affiliations:** 1 Department of Geology and Geophysics, University of Utah, Salt Lake City, Utah, United States of America; 2 Department of Atmospheric Sciences, University of Utah, Salt Lake City, Utah, United States of America; University of Vigo, Spain

## Abstract

Extra-tropical cyclones, such as 2012 Superstorm Sandy, pose a significant climatic threat to the northeastern United Sates, yet prediction of hydrologic and thermodynamic processes within such systems is complicated by their interaction with mid-latitude water patterns as they move poleward. Fortunately, the evolution of these systems is also recorded in the stable isotope ratios of storm-associated precipitation and water vapor, and isotopic analysis provides constraints on difficult-to-observe cyclone dynamics. During Superstorm Sandy, a unique crowdsourced approach enabled 685 precipitation samples to be obtained for oxygen and hydrogen isotopic analysis, constituting the largest isotopic sampling of a synoptic-scale system to date. Isotopically, these waters span an enormous range of values (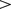
21‰ for 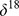
O, 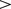
160‰ for 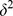
H) and exhibit strong spatiotemporal structure. Low isotope ratios occurred predominantly in the west and south quadrants of the storm, indicating robust isotopic distillation that tracked the intensity of the storm's warm core. Elevated values of deuterium-excess (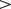
25‰) were found primarily in the New England region after Sandy made landfall. Isotope mass balance calculations and Lagrangian back-trajectory analysis suggest that these samples reflect the moistening of dry continental air entrained from a mid-latitude trough. These results demonstrate the power of rapid-response isotope monitoring to elucidate the structure and dynamics of water cycling within synoptic-scale systems and improve our understanding of storm evolution, hydroclimatological impacts, and paleo-storm proxies.

## Introduction

Addressing the challenge posed by extra-tropical cyclones requires a detailed understanding of how mid-latitidue weather patterns interact with the water and energy budgets of poleward moving storms [1], [2]. Information about the hydrologic and thermodynamic processes of these large storms is recorded as distinct isotopic signatures in the waters of contemporary hurricanes [3]–[10] as well as in isotopic proxies found in the prehistoric record [11]–[13]. The stable isotope ratios of oxygen, 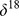
O [‰], and hydrogen, 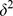
H [‰], (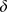 = R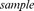/R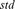-1 where R = ^2^H/^1^H or ^18^O/^16^O) obtained from cyclonic precipitation are thus informative tools that can aid in untangling the dynamic evolution of extra-tropical systems.

Early studies of isotope ratios in hurricanes [3], [4] found precipitation samples extremely depleted of heavy isotopes. Isotopic values well below the typical range of low and mid-latitude precipitation were attributed to the increased longevity, size, and thickness of clouds within cyclonic systems. This rainout effect is known to arise because the progressive loss of moisture coupled with temperature dependent equilibrium fractionation drives heavier isotopes to preferentially rainout, and the remaining vapor becomes increasingly depleted in the heavy isotopes [14]. Previous investigations into the isotopic composition of large storms have typically been limited in space or time, with samples collected at dense temporal intervals at a specific location (e.g. [9], [10]), across coarse spatial grids (e.g. 22 stations in the Eastern US [4]), or via aircraft transects of storms [7], [6]. Nonetheless, coherent spatial structures within cyclones have been observed, with isotope ratios decreasing radially inward toward the eyewall, and on the left side of landfall [5]–[7].

The observed patterns in isotopic ratios have been used to examine the hydrologic budget of large cyclones [7], [8], [10]. Isotopic analyses have revealed the energetic evolution of storm intensity, including high precipitation efficiencies and low condensation temperatures [5], [9] as well as the strength of vertical and radial circulation [8]. Changes in hurricane updraft velocities and upper level outflow alter hydrometeor descent paths and have been identified via stable isotope analysis of aircraft collected vapor [7]. Additional post-condensation processes including partial re-evaporation of falling precipitation and equilibration with surrounding vapor can further enrich hydrometeors before they reach the ground [15], [16], complicating interpretation of ground based precipitation composition. Specific characteristics of a storm's hydrologic budget, particularly moisture recharge via sea-spray, and vapor source conditions impart distinct isotopic signatures onto hurricane waters [8], [10]. These arise because strong surface gradients in humidity and elevated wind speeds increase kinetic fractionation more strongly in 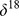
O relative to 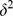
H, and the deuterium excess, 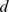 [‰] (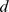 = 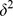
H−8
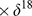
O), of recently evaporated moisture principally reflects conditions at the source of evaporation [26]. Therefore, a combination of multiple isotope tracers (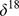
O and 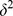
H) allows for assessment of both storm intensity and storm moisture sources.

**Figure 1:**
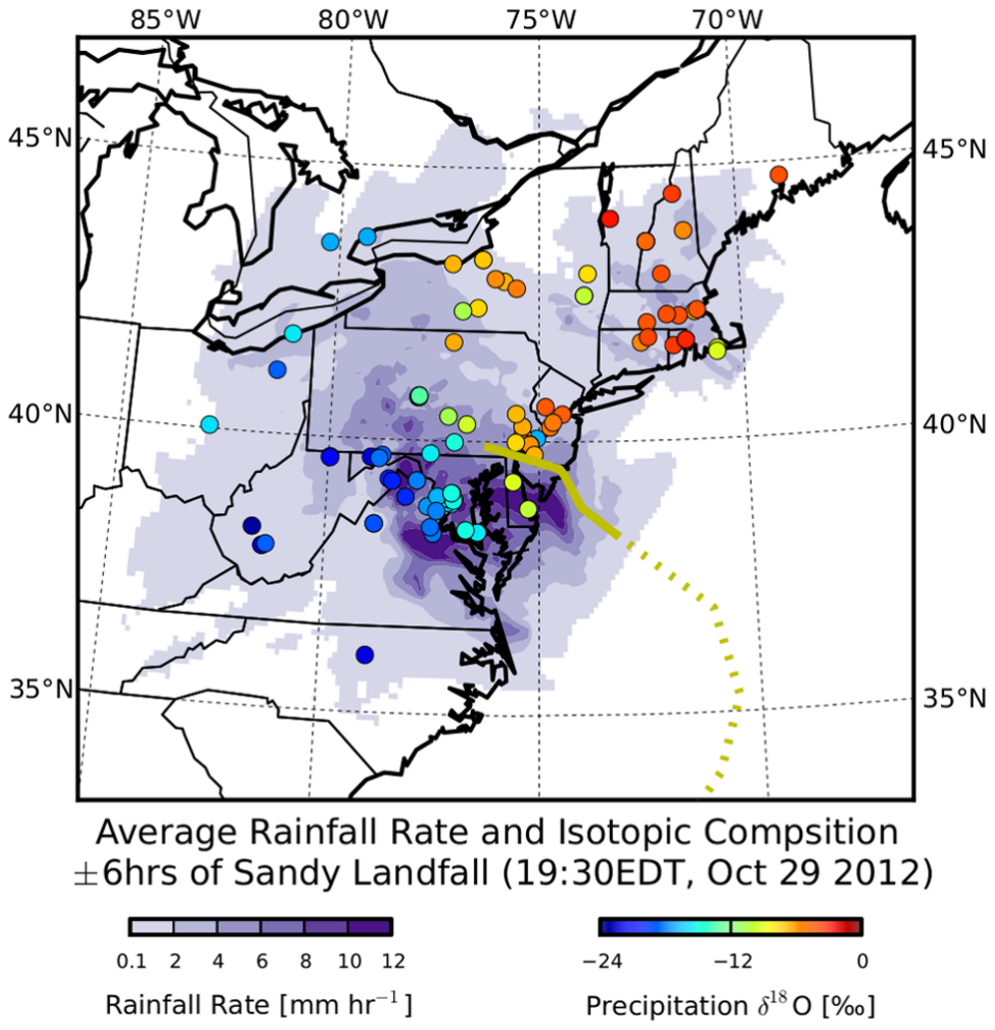
Snapshot of rainfall and 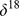
O isotope values during landfall of Superstorm Sandy. Storm track has landfall
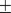
6hrs as solid line.

Contemporary records of cyclonic activity are relatively short compared to the timescale of multi-decadal climate variations that are known to influence the frequency and intensity of these large events [18]. Reconstruction of past climate regimes is critical to understating multi-decadal patterns in storm occurrences, and stable isotopic ratios preserved in tree rings [11], cave speleothems [12], and lake sediment cores [13] have been used to create paleoclimate records of cyclonic activity. Quantifying the determinants of the large spatial and temporal isotopic patterns in modern storms is needed for correct interpretation of these paleo-storm proxies. Finally, the highly unusual storm track of Sandy caused by a significant equatorward shifting of the jet stream in conjunction with the presence of a blocking anti-cyclone over the north Atlantic [19] demonstrates the complexity arising as synoptic scale systems transition from tropical to extra-tropical. As Sandy moved northward, the storm encountered a cold mid-latitude trough moving eastward over the central United States that caused large thermal asymmetries which ventilated the warm core of the storm [20], [21]. These atmospheric conditions resulted in an exceptionally large and complex extra-tropical cyclone which directly struck the major population centers along the U.S. eastern seaboard. Here, we present a unique crowdsourced dataset of precipitation stable isotope ratios for Superstorm Sandy that exhibit exceptional spatiotemporal structure (e.g. Figure 1) and assess the hydrologic patterns and processes reflected in these isotopic distributions.

## Methods and Materials

### Crowdsourced precipitation sampling

Precipitation samples presented here were obtained through a crowdsourcing approach, whereby the public at large was directly engaged to collect the water samples as the storm passed. On October 26, 2012, as Sandy was moving northeast off the coast of Florida, we began soliciting storm water sampling volunteers via science community email lists, Facebook, and Twitter. Information about the effort was further distributed through email forwards, science blogs and crowdsourcing sites. A blog was established to provide background information, documentation, instructions, and progress updates to participants (http://wateriso.utah.edu/waterisotopes/pages/data_access/sandy.html). We requested that samples be collected in a well-anchored receptacle placed in an unsheltered outdoor location, with collection every twelve hours, at or near 8AM and 8PM eastern time. Volunteers were instructed to store and ship samples in airtight containers, and to record and submit basic sample metadata, including information on sample locations, times, and phases.

Our study complies with all PLOS ONE ethical policies. Following the PLOS ONE guidelines, we make the following statements: No permits were required for the described study, which complied with all relevant regulations. Any rain samples collected from private property were collected directly and voluntarily by property owners. No protected species were sampled, nor was any animal husbandry, experimentation, or care/welfare, employed. No identifying information of individuals is reported in this work. No human subjects were studied and no institutional review board approval was needed. All participants provided written consent in the form of their emails volunteering to participate in the collecting effort. As outlined in the University of Utah Institutional Review Board (IRB, http://irb.utah.edu/), our study does not meet either the United States FDA or DHHS criteria for human subject research because no information was collected about the participants. We consulted with the University of Utah IRB and received confirmation that university IRB policies did not require review of our study.

Samples were shipped to our lab beginning in early November 2012, and in sum we received 685 samples from more than 125 volunteers. Following a preliminary visual quality screening that removed a small number of samples stored in containers that were not clean, poorly sealed, or had leaked, aliquots of each sample were transferred to septum-capped vials and analyzed for their H and O isotopic composition on a Picarro L2130-i analyzer. For each analysis approximately 1.2 microliters of water was injected into a heated vaporizer and then transferred to the cavity of the spectroscopic analyzer where isotopologue concentrations were determined by cavity ringdown spectroscopy [22]. Four injections of each unknown sample were made, and measurements of a suite of three laboratory reference waters (PZ: 16.9‰, 1.65‰; PT: −45.6‰, −7.23‰; UD: −123.1‰, −16.52‰; for 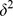
H and 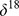
O, respectively) included in each analysis batch were used to correct raw data for sample-to-sample memory effects [23] and through-run drift. Data for all four injections were averaged to obtain uncalibrated sample values, and values for the UD and PZ waters were used to calibrate sample values to the VSMOW-SLAP reference scale using a two-point linear calibration. All samples were analyzed between November 7th and December 21st, 2012. Analytical precision (1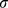) over this period was 0.32‰ for 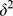
H and 0.04‰ for 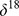
O (calculated as the standard deviation of the mean, calibrated PT values obtained in the 47 separate analysis batches).

**Figure 2:**
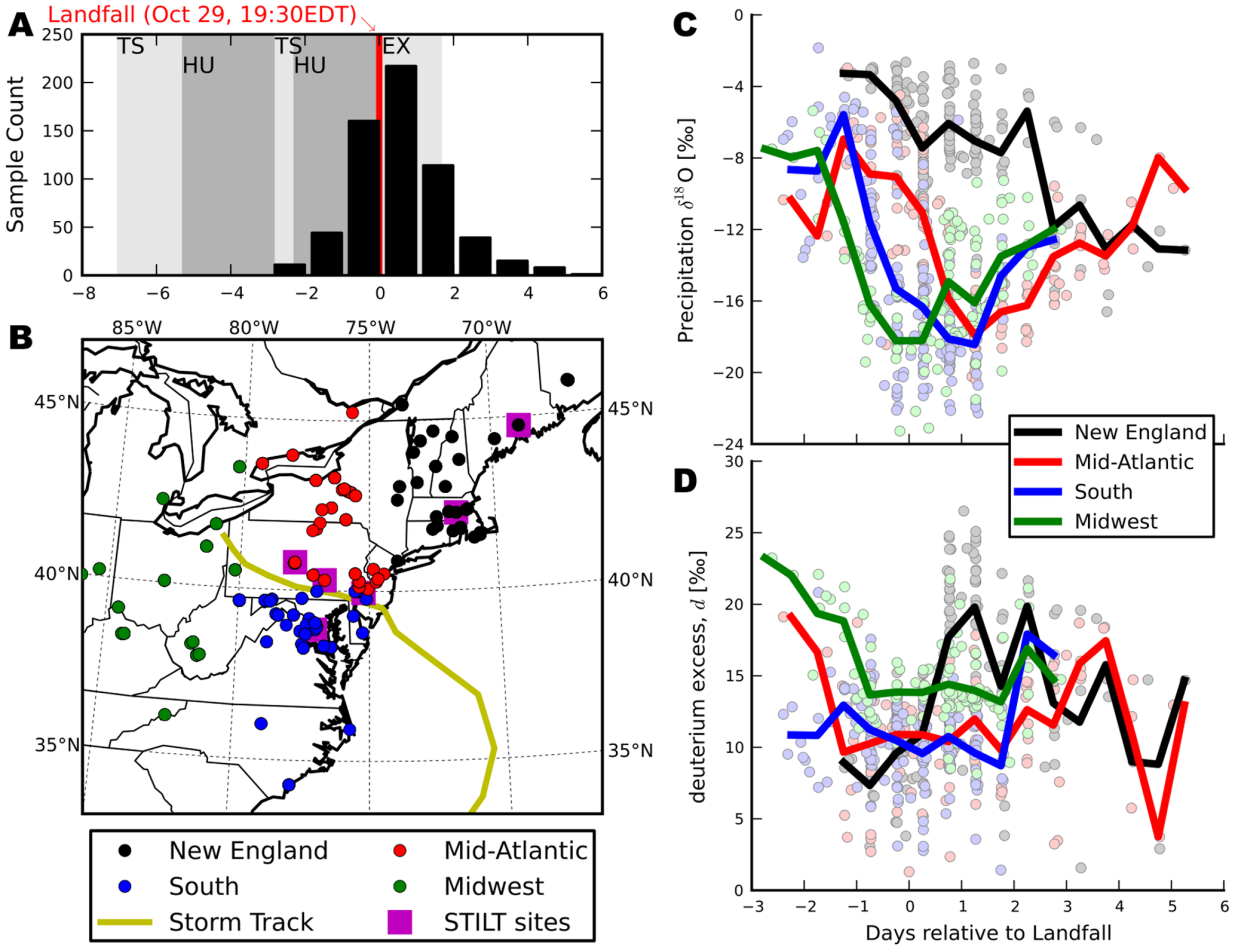
Spatial and temporal distribution of Sandy precipitation samples. (**A**) Histogram of the temporal distribution of collected precipitation isotope samples with NOAA classification of Sandy development: TS, Tropical Storm; HU, Hurricane; EX, Extra Tropical. (**B**) Spatial distribution of collected precipitation isotope samples by region (New England, Mid-Atlantic, Midwest, and South) with the Sandy storm track. Average regional precipitation (**C**) 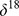
O and (**D**) 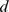-excess throughout the course of the storm color-coded by region.

**Figure 3:**
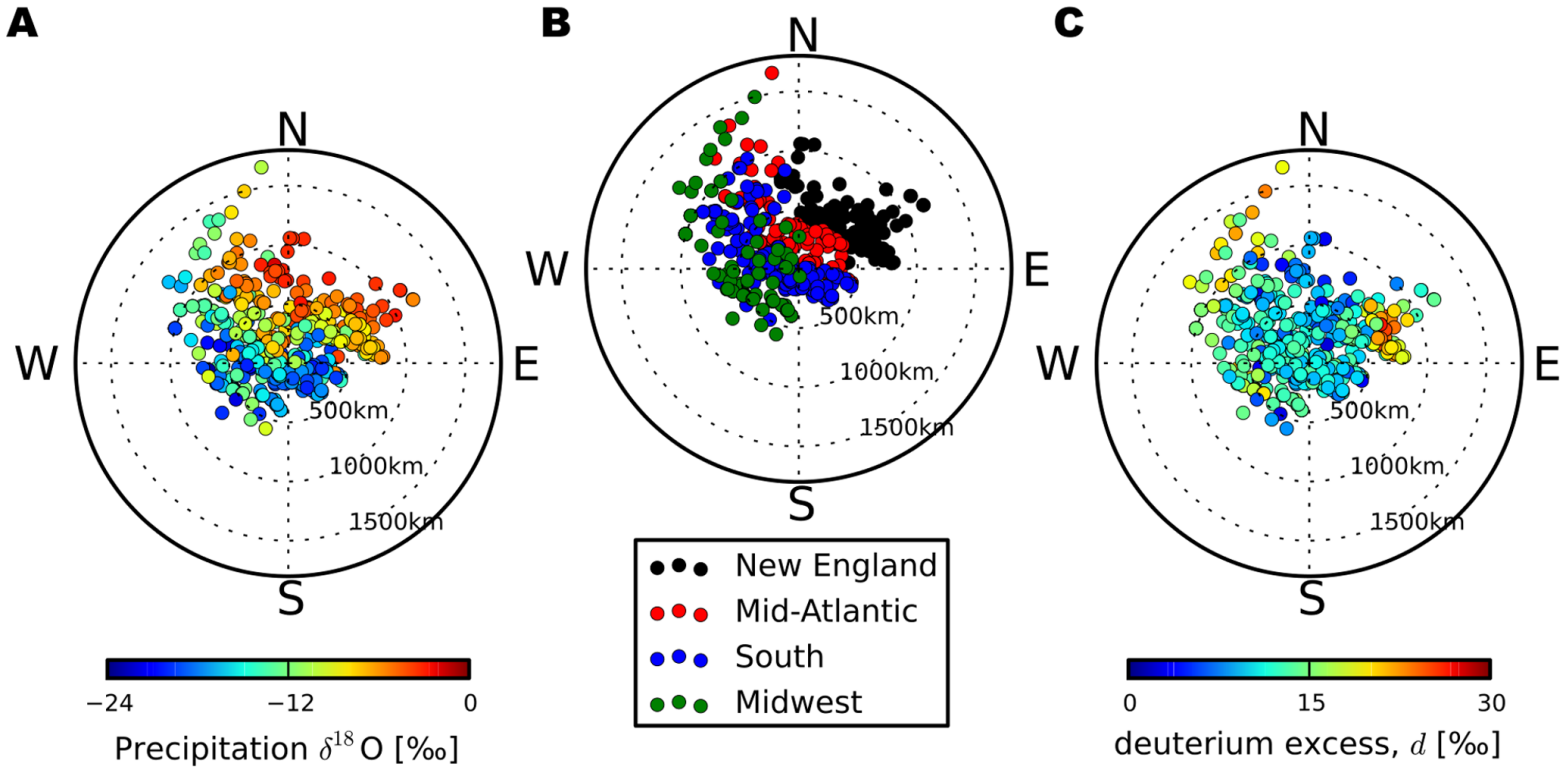
Sandy precipitation isotopic composition relative to storm center. Precipitation (**A**) 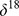
O and (**C**) deuterium-excess with data points positioned relative to the storm center at time of sample collection. (**B**) Samples are grouped by region as in Figure 1.

Measured 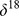
O and 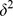
H values, as well as sample collection locations and times are published as a supplementary table (Dataset S1). Figures 2A and 2B show the temporal and spatial distribution of collected samples. Sample times are expressed here relative to landfall, which occurred at approximately 7:30PM EDT on October 29th, 2013 south of Atlantic City, New Jersey. For presentation, sites were divided into four geographic regions: New England (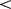
74°W), Mid-Atlantic (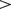
74°W & 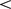
80°W & 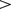
40°N), South (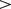
74°W & 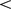
80°W & 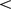
40°N), and Midwest, (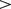
80°W). Shown in Figure 3 are the distance and bearing of each sample relative to the storm center at the time of collection, with samples grouped in Figure 3B by the same geographic regions. The storm track and stage classifications were obtained from the HURDAT2 Best Track Data, provided by the National Oceanic and Atmospheric Administration's (NOAA) National Hurricane Center (http://www.nhc.noaa.gov/data). Stage IV radar rainfall mosaics are from the NOAA National Center for Environmental Prediction (NCEP) [24]. All maps were generated with the Basemap extension of the Matplotlib [15] Python library.

### Lagrangian back-trajectory assessment

Atmospheric trajectory analysis provides constraints on the interpretation of precipitation isotopic composition [26], [27]. Here we use the Stochastic Time-Inverted Lagrangian trajectory (STILT) model [28] to infer moisture source conditions and transport for Sandy precipitation samples. The STILT model is a state of the art Lagrangian dispersion model used to trace the transport of scalars in the atmosphere by computing the influence of upstream regions on a modeled receptor via the simulation of many particles backward through time. Unlike single trajectory models adopted in previous isotopic studies, STILT represents atmospheric transport with an ensemble of particles, with each particle moving quasi-stochastically in response to the turbulence strength [29]–[31]. Particles are advected based on 12-km gridded wind fields from NOAA-NCEP's North American Mesoscale Model final analysis product (NAM FNL) [32], with turbulence treated as a Markov chain process. Temperatures and specific humidity at the particle locations are also extracted from NAM-FNL.

**Figure 4:**
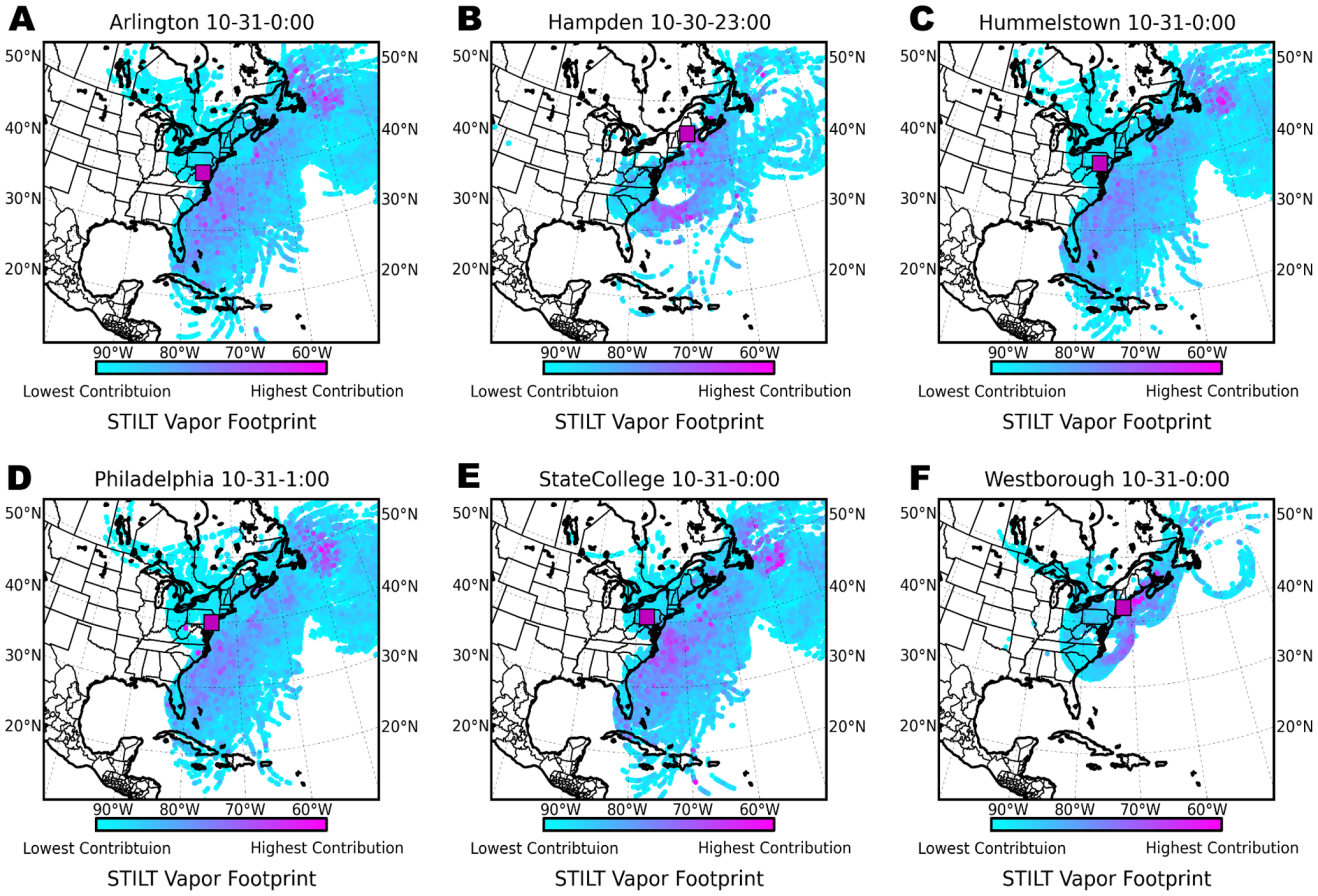
Estimates of vapor source regions. (**A–F**) Back trajectory estimates from the STILT model of regions contributing moisture to the atmosphere above six locations where precipitation samples were collected on October 31st 2013, 00 hrs EDT.

STILT back trajectories at the six locations shown in Figure 2 were carried out every twelve hours, with simulations going backward 120 hours in fifteen minute timesteps, starting on Oct 29th around 12:00PM. The six STILT sites used in this study correspond to locations where a large number of samples were collected and were located at: Arlington VA (38.8518N, 77.0524W), Hampden ME (44.7344N, 68.8349W), Hummelstown PA (40.3113N, 76.7214W), Philadelphia PA (39.9507N, 75.2173W), State College PA (40.8150N, 77.8830W), and Westborough MA (42.2718N, 71.5459W). The STILT model estimates the unit flux footprint, 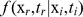, for a receptor at location 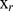 and time 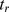 from source area 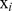 and time 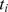 by counting the fraction of Lagrangian particles within the turbulently mixed planetary boundary layer at that time and place. This footprint quantifies the contribution of a source of unit strength at a particular location to the final scalar mixing ratio arriving at the receptor. A normalized flux-weighted vapor footprint is then calculated as 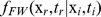  =  
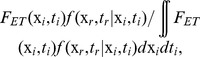
 where the evapotranspiration flux, 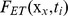 is approximated by the time derivative of each particle's specific humidity. Ensembles of 5 particles were released every 15 minutes and tracked for 120 hours into the past. Particles were released from heights of 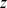 = 500 m, 3500 m, 6500 m, 9500 m, 12500 m, and 15500 m above ground level with 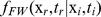 values estimated based on data from all heights (note, we normalize across all heights such that: 
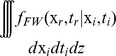
 = 1). Figure 4 shows an example of STILT 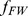 values at the six STILT locations for the simulation block starting October 31st at 00∶00 hrs.

These back trajectories enable estimation of flux-weighted source vapor temperature and relative humidity, from which source isotopic composition is modeled. We calculate the surface normalized flux-weighted source vapor relative humidity, 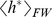 = 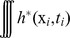 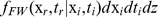, for each set of back trajectories. In a similar manner we also calculate the flux-weighted source vapor surface temperature, 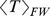, with angle brackets denoting averaging in space and time along the ensemble of particle pathways. Given evaporation conditions, 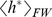, and 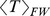, source vapor isotopic composition is modeled following [17] as 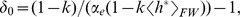 where 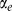 is the temperature dependent equilibrium fractionation factor [25], and 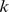 is the kinetic fractionation factor (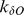 = 6.2‰, 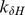 = 5.5‰ [34]). As a first order approximation, the rainout fraction of a given air parcel (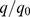) can be expressed via Rayleigh distillation as 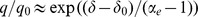 [8], [14]. This simplified approach enables estimation of rainout efficiency (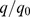) though the assumption of instantaneous removal of condensed liquid, with 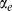 calculated iteratively based on the dew point of the rising air parcel [8].

## Results and Discussion

### Isotopic patterns in Superstorm Sandy

The collected precipitation samples exhibited strong spatiotemporal patterns in their stable isotope composition. The average 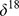
O value across all samples was −12‰, with regional means of −6.8‰ (New England), −12.7‰ (Mid-Atlantic), −14.8‰ (South), and −14.9‰ (Midwest). As Sandy moved northwest, precipitation became substantially more depleted of heavy isotopes (Figure 2C), with decreases of 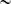
10‰ occurring in the Midwest before landfall, in the South and Mid-Atlantic during landfall, and in New England continuing well after landfall. Excluding the Midwest, declines in 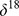
O became less steep and occurred later as Sandy lost intensity (HURDAT2 classifications shown in Figure 2A). The lowest 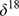
O values were observed in the Midwest immediately around landfall (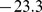
‰), and are likely associated with a mid-latitude cold front moving eastward when the storm made landfall. After landfall, as Sandy moved west towards the Great Lakes, 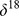
O values rose again in the Mid-Atlantic, South, and Midwest regions. New England 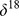
O values were consistently higher than those in other regions throughout the course of the storm and exhibited a weak decline following landfall with no subsequent increase in 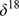
O values. Overall, a strong north-east to south-west gradient existed in Sandy 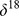
O values relative to the storm's core (Figure 3A), with an approximate decrease of 2.4‰ per 100 kilometers observed. A large portion of the 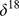
O values observed in precipitation associated with Sandy are lower the typical mid-Atlantic precipitation and the direction of the 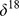
O gradient observed during Sandy is opposite to typical latitudinal gradient [35] in precipitation isotopic composition.

Deuterium excess values of precipitation falling during Sandy also demonstrate strong spatiotemporal structure. The storm average 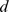 for precipitation was 12‰, with regional means of 13.9‰ (New England), 11.2‰ (Mid-Atlantic), 10.6‰ (South), and 14.7‰ (Midwest). Though Midwestern 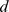 values were higher on average, the highest 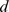 values (26.5‰) were collected in New England after Sandy made landfall. From one day before landfall through two days afterward, 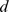 values in the Mid-Atlantic, South, and Midwest regions were uniform within each region, with the Midwest slightly higher (Figure 2D). Early precipitation collected in the Midwest exhibited high 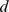 values, with these waters most likely associated with the mid-latitude trough moving eastward over this region. Excluding these early anomalous samples, the major cluster of elevated 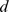 values was found in New England (Figure 3C), and occurred there only after the storm center had moved off to the west. The abrupt increase in 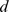 values in the New England region one day after landfall is substantially different than the 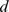 trends from all other regions. Distance to the storm center was more strongly correlated to 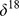
O values (Pearson's 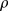 = 0.42) than to 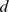 values (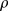 = 0.34).

Local rainfall amounts averaged over the duration represented by each precipitation sample demonstrate congruence with collected 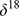
O and deuterium excess values. The most intense precipitation occurred as Sandy made landfall (Figure 1), with the largest rainfall rates (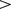
10 mm hr^−1^) occurring South of the storm center. This region of strong precipitation was also where a majority of the extremely low 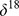
O values were observed. Samples from the southern region had an average rainfall rate of 2.5 mm hr^−1^, much higher than the storm-wide average of 1.0 mm hr^−1^. Throughout the storm, 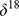
O values were weakly anti-correlated with higher rainfall rates (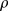 = −0.15).

### Extra-tropical cyclonic evolution

**Figure 5:**
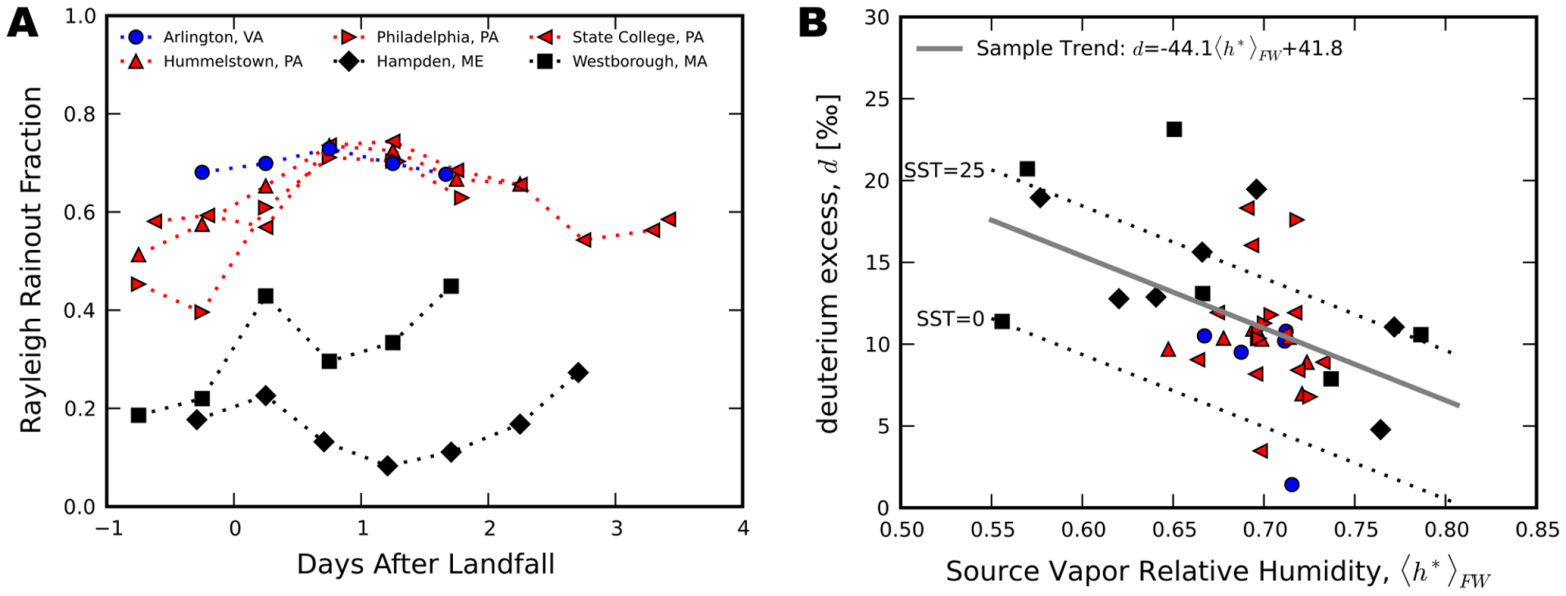
Isotopic signatures of Sandy intensity and interaction with dry continental air. (**A**) Rayleigh rainout fraction at STILT sites based on change in isotopic composition from estimated source moisture to collected precipitation sample. (**B**) Deuterium-excess at STILT trajectory locations and times (symbols) as a function of surface normalized source vapor relative humidity, 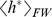. The linear trend fit to collected samples (gray line) falls between 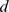 values expected from vapor with sea surface temperatures (SST) of 0°C and 25°C (dotted lines). Symbols are the same in **A** and **B** and colors match the regions of Figure 2.

The coherent spatiotemporal isotopic patterns within Sandy's precipitation can be related to the thermodynamic processes occurring as the storm transitioned from tropical to extra-tropical. Highly efficient cyclonic dehydration within the Sandy warm core likely occurred during and immediately after landfall as demonstrated by the extremely low 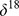
O values in the South, Mid-Atlantic, and Midwest regions. Condensation removes latent heat input into the storm during surface evaporation, and efficient dehydration of moist air corresponds to strong energy transport. The uniform 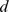 values in these regions for days −1 to +2 (Figure 2C) suggest that there were not major changes in moisture source within each region, thus differences in isotope values are principally a result of variation in storm rainout efficiencies. Based on STILT results, source vapor 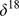
O is estimated at −11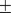.2‰ during this period, when the collected rainfall composition dropped from −6‰ to −18‰ across these regions. Rayleigh rainout of this source vapor of approximately 20% is needed to arrive at a final precipitation composition of −6‰, while rainout of approximately 60% is required to arrive at final precipitation compositions of −18‰ (Figure 5A). Though this approach, we estimate that the precipitation efficiency in Sandy core peaked between one and two days after landfall, after which the storm's energy subsided.

These finding are consistent with microwave soundings of the warm-core structure within Sandy, which found this storm to be about 4

K cooler than other comparable storms [20]. These cooler final condensation temperatures within the upper core most likely caused high rainout efficiencies and extremely low 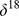
O values. The equilibration and evaporation of falling precipitation also alters 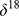
O values; however, sensitivity tests on numerical simulations of the isotopic budget of hurricanes have demonstrated that the factors influencing rainout fraction (e.g. higher condensation altitudes and lower condensation temperatures) have the greatest impact on the low 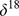
O values that occur in the stratiform region 50–250 km from the storm center [8]. Within Sandy, the isotopically light precipitation values consistently track the leftward side of the storm as Sandy moved northward. This region near, but not at the storm center, is where intense precipitation with the longest overland trajectories occurred, and therefore where surface to atmosphere transfer of energy was most efficient.

As Sandy moved northwest after landfall, elevated deuterium excess values found in the New England portion of the storm indicate complex interaction with mid-latitude weather patterns. Merlivat and Jouzel [17] demonstrated that high values of 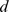 (e.g 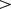
15‰) can only occur in calm conditions if boundary layer relative humidity over oceanic vapor source regions is very low (i.e. 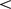
60%), but can also occur under more humid conditions if wind speeds are significantly higher or, as noted by Gedzelman et. al. [8], if substantial evaporation comes from sea spray. Conditions necessary to create the 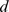 values 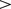
20‰ observed in New England require off-shore evaporation into a significant block of relatively dry air. The most probable source of this dry air is the entrainment of air from the mid-latitude trough that collided with Sandy and was drawn counter-clockwise around the system, over the Atlantic, and into New England (Figure 4B and 4F).

During this period of Sandy's evolution, the storm's outer bands transported dry, continental air over the Atlantic, where evaporation at low relative humidities (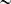
55%-65%), and possibly elevated wind speeds, most likely created boundary layer vapor with high 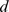 values that later precipitated in the northeast. Based on the STILT back-trajectory model, precipitation samples with elevated deuterium excess values were associated with lower relative humidity values in vapor source regions (Figure 5B). The linear trend fit to sample 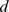 as a function of source relative humidity is consistent with modeled (following [17]) footprint-weighed 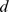 values based on surface temperature and relative humidity. Deviations are likely associated with missing processes: i.e., the effects from wind-speed, sea surface temperature, and sea spray. However, the broad asymmetrical nature of observed 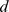 values clearly indicate influence of a relatively dry continental air mass in the precipitation collected in the New England region. This suggest that continued evaporation driven by low humidities in the air derived in part from the mid-latitude trough sustained latent energy inputs into Sandy's outer bands. This late stage energy influx likely contributed to the large size and prolonged intensity of Superstorm Sandy during its extra-tropical stage.

## Conclusion

The new dataset published here constitutes the largest synoptic scale isotope study of precipitation to date and demonstrates strong spatiotemporal patterns in both the 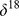
O and deuterium excess values of Superstorm Sandy precipitation. The extremely low 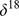
O values collected southwest of the storm center indicate the region of intense precipitation where rainout efficiency was highest. This region of highly 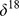
O depleted rainfall tracked the storm center, with the decreasing regional minimum in 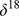
O reflecting waning storm intensity. Analysis of patterns in deuterium excess demonstrate that as Sandy moved north, interaction with a mid-latitude cold front brought dry continental air into the outer bands of the system, which then gained moisture over the Atlantic and precipitated out over New England.

The extra-tropical transition of Superstorm Sandy and incorporation of the mid-latitude trough created a complex cyclonic structure with a distinct isotopic signature. The extensive spatial and temporal distribution of these collected storm waters provide insight into difficult-to-monitor thermodynamic processes, such as energy transport within the cyclonic core, open-ocean evaporation processes, and interaction with extra-tropical weather patterns. Information gained here on mechanisms driving patterns of spatiotemporal isotopic variation in a major extratropical cyclone will improve interpretation of paleoclimate proxies and aid in constraining the hydrologic and energy budgets of past and future cyclones.

## Supporting Information

Dataset S1 **Precipitation isotope composition.** Precipitation sample location, time, and isotopic composition are published as a comma separated value file ‘DatasetS1.csv’.
(CSV)
